# Heart rate variability and peripheral nerve conduction velocity in relation to blood lead in newly hired lead workers

**DOI:** 10.1136/oemed-2018-105379

**Published:** 2019-03-30

**Authors:** Cai-Guo Yu, Fang-Fei Wei, Wen-Yi Yang, Zhen-Yu Zhang, Blerim Mujaj, Lutgarde Thijs, Ying-Mei Feng, Jan A Staessen

**Affiliations:** 1 Department of Cardiovascular Sciences, University of Leuven, Leuven, Belgium; 2 Department of Endocrinology, Beijing Lu He Hospital and Key Laboratory of Diabetes Prevention and Research, Capital Medical University, Beijing, China; 3 Institut Universitaire de Médecine Sociale et Préventive, University of Lausanne, Lausanne, Switzerland; 4 Cardiovascular Research Institute Maastricht, Maastricht University, Maastricht, The Netherlands

**Keywords:** lead, neurophysiology, cardiovascular, materials, exposures and occupational groups, healthcare workers

## Abstract

**Objectives:**

Previous studies relating nervous activity to blood lead (BL) levels have limited relevance, because over time environmental and occupational exposure substantially dropped. We investigated the association of heart rate variability (HRV) and median nerve conduction velocity (NCV) with BL using the baseline measurements collected in the Study for Promotion of Health in Recycling Lead (NCT02243904).

**Methods:**

In 328 newly hired men (mean age 28.3 years; participation rate 82.7%), we derived HRV measures (power expressed in normalised units (nu) in the high-frequency (HF) and low-frequency (LF) domains, and LF/HF) prior to long-term occupational lead exposure. Five-minute ECG recordings, obtained in the supine and standing positions, were analysed by Fourier transform or autoregressive modelling, using Cardiax software. Motor NCV was measured at the median nerve by a handheld device (Brevio Nerve Conduction Monitoring System, NeuMed, West Trenton, NJ, USA). BL was determined by inductively coupled plasma mass spectrometry.

**Results:**

Mean BL was 4.54 µg/dL (IQR 2.60–8.90 µg/dL). Mean supine and standing values of LF, HF and LF/HF were 50.5 and 21.1 nu and 2.63, and 59.7 and 10.9 nu and 6.31, respectively. Orthostatic stress decreased HF and increased LF (p<0.001). NCV averaged 3.74 m/s. Analyses across thirds of the BL distribution and multivariable-adjusted regression analyses failed to demonstrate any association of HRV or NCV with BL.

**Conclusions:**

At the exposure levels observed in our study, autonomous nervous activity and NCV were not associated with BL.

**Trial registration number:**

NCT02243904

**Key messages**

**What is already known about this subject?**

- Previous studies relating nervous system activity to lead exposure have limited relevance because of substantially lower current environmental and occupational exposure.
- At high exposure levels in an occupational context, lead is neurotoxic, leading to cardiac autonomic dysfunction and reduced peripheral nerve conduction velocity.

**What are the new findings?**

- In 328 newly hired workers prior to chronic occupational lead exposure, the geometric mean blood lead concentration was 4.54 μg/dL (IQR 2.60–8.90).
- At these exposure levels, there was no association of heart rate variability with blood lead.
- These results were consistent irrespective of body position or the way heart rate variability was analysed in the frequency domain.
- There was no association between motor nerve conduction velocity assessed at the median nerve and blood lead.

**How might this impact on policy or clinical practice in the foreseeable future?**

- At current environmental and occupational exposure levels in the USA, lead may not be a cause of central autonomous or peripheral nervous dysfunction.
- As occupational safety and health regulatory agencies in North America, Europe and Australasia are proposing more stringent workplace limits for lead, this study may provide valuable information on blood lead levels that may not cause adverse health effects associated with occupational lead exposure.

## Background

Almost two decades ago, Araki and coworkers reviewed 102 articles on the effects of lead exposure on the peripheral, central and autonomous nervous system in workers.^1^ They reported that the reduction in the peripheral nerve conduction velocity, together with effects on postural balance and ECG heart rate variability, occurs at a mean blood lead concentration of 30 – 40  µg/dL. 1 A major limitation of the previous studies is that they have limited relevance in view of the current environmental exposure to lead. Indeed, in the USA, the National Health and Nutrition Examination Survey (NHANES) documented a progressive decline in the geometric blood lead concentration over time. Among adults, mean blood lead Among adults, mean blood levels decreased from 13.1  µg/dL in NHANES II (1976 – 1980)^2 3^ to 1.2 – 2.76  µg/dL in NHANES III (1988 – 1994),^2 3^ and to 1.64  µg/dL in NHANES IV (1999 – 2002) and in later examination cycles.^4 5^ Furthermore, a PubMed search without limitation of language or publication date and using as search terms ‘ lead exposure’ in association with ‘ heart rate variability ’ or ‘ sympathetic activity’ or ‘ parasympathetic activity’ or ‘ nerve conduction velocity ’ revealed that few relevant articles were published since 2000,^6–15^ and most publications focused on potential neurotoxic mechanisms in an experimental setting.^10–15^ Against this background, our aim was to investigate the association of heart rate variability and peripheral nerve conduction velocity with blood lead at low exposure levels, using the baseline measurements collected in the ongoing Study for Promotion of Health in Recycling Lead (SPHERL; NCT02243904) in newly hired workers prior to occupational lead exposure.^16^

## METHODS

### Study participants

SPHERL complies with the Helsinki declaration for investigations in humans. SPHERL is a longitudinal study of newly hired lead workers at battery manufacturing and lead recycling plants in the USA.^16^ From 1 May 2015 until 19 September 2017, a total of 556 men applied for a job and underwent a pre-employment physical examination, at which whole blood lead was measured as part of the routine health check-up put in place prior to employment. These pre-employment data are not part of the SPHERL study. Of 556 men who underwent this pre-employment assessment, all were invited to participate in SPHERL; 460 provided written informed consent and underwent the SPHERL baseline examination (participation rate, 82.7%). Of those, 424 had their ECG recorded and peripheral nerve conduction velocity and blood lead measured. We excluded 96 workers from the current analysis of the SPHERL baseline data, because of previous occupational exposure to lead (n=41), because the ECG was of insufficient quality (n=40), or because continuously distributed variables were more than 3 SDs above the mean in the whole study population (n=15). However, no worker was excluded because of a high blood lead concentration. Thus, the number of workers statistically analysed totalled 328.

### Clinical measurements

Blood pressure was the average of five consecutive auscultatory readings obtained with a standard mercury sphygmomanometer after the workers had rested for 5 min or longer in the sitting position.^16^ Mean arterial pressure was diastolic pressure plus one-third of the difference between systolic and diastolic pressure. High blood pressure was a level of ≥140 mm Hg systolic, or ≥90 mm Hg diastolic, or use of antihypertensive drugs. Body mass index was body weight (kg) divided by height squared (m^2^). The umbilicus and greater trochanter were the landmarks for measuring waist and hip circumference. Study nurses administered validated questionnaires, inquiring about each worker’s medical history, occupations, exposure to heavy metals, smoking and drinking habits, intake of medications and lifestyle.

### Biochemical measurements

Venous blood samples were obtained after 8–12 hours of fasting. Blood lead levels were determined on whole blood by inductively coupled plasma mass spectrometry at an analytical laboratory certified for blood lead analysis in compliance with the provisions of the OSHA Lead Standard, 29CFR 1910.1025 (Occupational Safety and Health Administration (www.osha.gov)). Prior to analysis, the specimens were digested with nitric acid and spiked with an iridium internal standard. The detection limit was 0.5 µg/dL. Serum total and high-density lipoprotein (HDL) cholesterol and blood glucose were measured by automated enzymatic methods and serum insulin by ELISA. The online supplementary material provides detailed information on the accuracy and the repeatability of the blood lead measurements as well as on the performance of the laboratory in proficiency testing for lead and the other biochemical analytes.

10.1136/oemed-2018-105379.supp1 Supplementary file 1

### Heart rate variability

At enrolment, the study nurses record a standard 12-lead ECG by means of the paperless Cardiax device. Heart rate variability was measured from 5 min ECG recordings in supine and standing positions, using the Cardiax software, V.4.14.0 (International Medical Equipment Developing, Budapest, Hungary). The Cardiax software allows exporting all ECG measurements into an Excel workbook, which was subsequently imported into SAS V.9.4, using standardised programming statements, thereby excluding any observer-induced bias. The software computes the power spectrum in the frequency domain by fast Fourier transform and by autoregressive modelling (online supplementary material) and provides the low-frequency (0.04–0.15 Hz) and high-frequency (0.15–0.40 Hz) components of heart rate variability in milliseconds and the low to high frequency ratio. Normalised units of low and high-frequency power were calculated as the low and high-frequency power divided by the difference (total power − very-low-frequency power) ×100. Heart rate variability reflects the activity of the autonomous nervous system. Efferent vagal activity is a major contributor to high-frequency power. The interpretation of low-frequency power is more controversial and reflects sympathetic modulation or both sympathetic and parasympathetic activity. Total frequency power decreases during sympathetic activation, whereas the reverse occurs during vagal activation.^17^

### Peripheral nerve conduction velocity

The study nurses used a handheld device and accompanying software (Brevio Nerve Conduction Monitoring System, NeuMed, West Trenton, NJ, USA) to stimulate the median nerve at a gradually increasing voltage until the maximum compound motor action potential of the short thumb abductor muscle was reached. Nerve conduction velocity was measured in the workers’ right and left hands. To check the quality of the nerve conduction velocity, we randomly selected 40 workers and produced the Bland and Altman statistics,^18^ comparing the velocity at the left and right arms (see online supplementary figure S1). The bias (right minus left side) was 0.014 m/s (p=0.83). The repeatability coefficient was 0.82.

### Data analyses

Database management and statistical analyses were done using SAS V.9.4 software (Cary, NC, USA). The flow and quality control of the data are described in the published protocol.^16^ Departure from normality was evaluated using Shapiro-Wilk statistic. Skewness and kurtosis were computed as the third and fourth moments of the mean divided by the cube of the SD. We applied a logarithmic transformation to normalise the distributions of total power, the low to high frequency ratio in the supine and standing positions, the orthostatic changes in all heart rate variability measurements and blood lead and serum insulin. The central tendency (spread) of normally distributed variables was represented by the arithmetic (SD) or geometric (IQR) mean. To compare means and proportions, we applied a t-statistic or analysis of variance, as appropriate, and the χ^2^ (with right symbol and format in the on-line system) statistic or Fisher’s exact test, respectively.

In exploratory analyses, we assessed heart rate variability and nerve conduction velocity across thirds of the blood lead distribution or by using unadjusted linear regression. P values for trend were computed by the χ^2^(with right symbol and format in the online system) statistic for categorical variables or by regressing continuously distributed variables on a design variable identifying the thirds of the blood lead distribution with as values 1, 2 or 3. We used stepwise regression analysis with the p value for covariables to enter and stay in the models set at 0.15. The covariables considered were age, body mass index, waist to hip ratio, mean arterial pressure, heart rate, current smoking and drinking, history of diabetes and kidney disease, treatment of hypertension, serum insulin levels (logarithmically transformed) and the total to HDL cholesterol ratio. Significance was a two-tailed α-level of 0.05 or less.

## Results

### Characteristics of participants

Table 1 lists the characteristics of the participants in all workers combined and after stratification by thirds of the blood lead distribution. The 328 newly hired workers included 149 Whites (45.4%), 155 Hispanics (47.3%), 12 Blacks (3.7%), 3 Asians (0.9%) and 9 workers of mixed ethnic descent (2.7%). Among 132 workers reporting current alcohol intake drinkers (40.2%), the median alcohol consumption was 4.8 g/day (IQR 1.8–9.3 g/day); 66 workers (50%) reported an alcohol consumption of 5 g/day or more.

**Table 1:** Characteristics of the workers

Characteristic	All workers	Stratified by thirds of the blood lead distribution			
		<3.1 µg/dL	3.1–7.0 µg/dL	>7.0 µg/dL	P value
Number in category	328	106	112	110	
Number (%) with characteristic					
Current smoking	96 (29.3)	25 (23.6)	38 (33.9)	33 (30)	0.24
Current alcohol intake	132 (40.2)	38 (35.9)	52 (46.4)	42 (38.2)	0.24
High blood pressure	34 (10.4)	9 (8.5)	18 (16.0)	7 (6.4)*	0.045
Treated hypertension	21 (6.4)	7 (6.6)	11 (9.8)	3 (2.7)*	0.10
Diabetes mellitus	7 (2.1)	2 (1.9)	3 (2.7)	2 (1.8)	0.89
History of CV disease	17 (5.2)	8 (7.5)	5 (4.5)	4 (3.6)	0.40
Mean of characteristic					
Age (years)	28.3±10.2	28.7±10.1	29.8±11.5	26.4±8.7*	0.090
Body mass index (kg/m^2^)	28.6±6.2	29.3±6.5	29.1±6.3	27.5±5.6	0.027
Waist to hip ratio	0.97±0.07	0.97±0.07	0.98±0.07	0.95±0.07*	0.032
Systolic pressure (mm Hg)	120.6±9.7	120.5±9.2	122.2±10.0	119.1±9.5*	0.27
Diastolic pressure (mm Hg)	80.7±7.9	80.2±8.2	81.7±8.0	80.2±7.5	0.95
Mean arterial pressure (mm Hg)	94.0±7.7	93.7±7.9	95.2±7.8	93.1±7.4	0.62
Total cholesterol (mg/dL)	169.4±36.9	172.3±37.0	174.0±39.2	162.1±33.6*	0.039
HDL cholesterol (mg/dL)	45.9±10.5	44.3±9.6	46.5±10.0	47.0±11.7	0.058
Total to HDL cholesterol ratio	3.88±1.21	4.08±1.29	3.90±1.15	3.66±1.16	0.011
Insulin (μIU/mL)	7.3 (3.9–12.7)	7.5 (4.5–13.0)	7.4 (4.0–13.0)	6.8 (3.4–11.8)	0.37
Blood lead (μg/dL)	4.54 (2.50–8.30)	1.81 (1.30–2.60)	4.60 (3.70–5.40)***	11.0 (8.90–12.2)***	<0.0001

Average values are arithmetic (±SD) or geometric means (IQR). High blood pressure was a level of ≥140 mm Hg systolic, or ≥90 mm Hg diastolic, or use of antihypertensive drugs. Mean arterial pressure was diastolic pressure plus one-third of the difference between systolic and diastolic pressure. P values are for linear trend across thirds of the blood lead distribution.

Significance of the difference with the adjacent left column: *P≤0.05; ***P≤0.001.

CV, cardiovascular; HDL, high-density lipoprotein.

**Figure 1:**
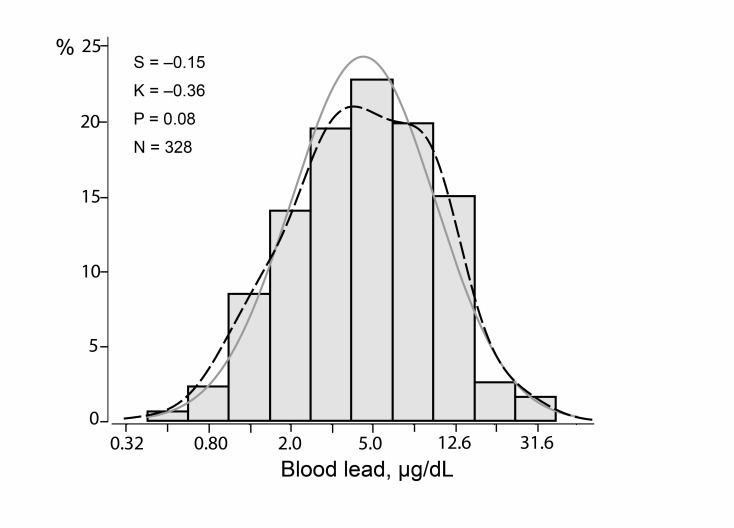
Distribution of logarithmically transformed blood lead. S, K and M indicate the coefficients of skewness and kurtosis and the geometric mean, respectively. The solid and dotted lines represent the normal and kernel density distributions. The p value is for departure of the actually observed distribution from normality according to Shapiro-Wilk statistic.

At the pre-employment physical examination, the geometric mean blood lead concentration was 2.47 µg/dL (IQR 2.00–3.00 µg/dL). Of the 328 workers, 286 (87.2%) were involved in the manufacturing process and were therefore exposed, whereas 42 (12.8%) employees had clerical or warehouse tasks or were sales representatives without lead exposure. The geometric mean lead concentration in inhaled air in the lead-exposed workers according to National Institute for Occupational Safety and Health standards (NIOSH; https://www.cdc.gov/niosh/topics/lead/limits.html) was 11.3 µg/m^3^ (IQR 6.0–21.0 µg/m^3^). Lead in the air was not monitored in departments of the plants, where there was no lead exposure. The first assessment of blood lead for the current study, done simultaneously with the heart rate variability and peripheral nerve conduction velocity measurements, was performed 21 days (IQR 10–31 days) after workers had started employment. At this time point, the geometric mean blood lead concentration was 4.54 µg/dL (IQR 2.50–8.30 µg/dL; figure 1). Across the lead categories, the prevalence of high blood pressure, body mass index, the waist to hip ratio, total cholesterol and the total to HDL cholesterol ratio all decreased (p≤0.045; table 1).

### Heart rate variability

Table 2 provides the heart rate variability measurements derived by Fourier transform in all workers and after stratification by thirds of the blood lead distribution. The corresponding measurements derived by autoregressive modelling appear in online supplementary table S1. Analyses across thirds of the blood lead distribution did not reveal any significant trend in heart rate or in the measures of heart rate variability as derived by Fourier transform (table 2) or by autoregressive modelling (see online supplementary table S1). These findings were consistent for observations made in the supine and standing positions as well as for the orthostatic changes in heart rate and heart rate variability.

**Table 2:** Heart rate variability by Fourier transform

Characteristic	All workers	Stratified by thirds of the blood lead distribution			
		<3.1 µg/dL	3.1–7.0 µg/dL	>7.0 µg/dL	P value
Number in category	328	106	112	110	
Supine position					
Heart rate (beats/minute)	65.4±10.8	66.9±11.3	65.2±10.2	64.3±10.6	0.080
Total power (m/s^2^)	1621 (791 to 3088)	1492 (789 to 2698)	1547 (745 to 3038)	1828 (891 to 3306)	0.14
Low-frequency power (nu)	50.5±15.6	52.6±15.9	49.0±14.7	50.0±16.1	0.22
High-frequency power (nu)	21.1±11.8	20.7±11.6	22.4±12.6	20.1±11.0	0.72
Low to high frequency ratio	2.63 (1.66 to 4.07)	2.80 (1.68 to 4.61)	2.50 (1.56 to 3.67)	2.69 (1.87 to 4.09)	0.67
Standing position					
Heart rate (beats/minute)	77.8±12.7	79.4±13.5	78.0±12.5	76.3±12.0	0.069
Total power (m/s^2^)	1445 (818 to 2658)	1458 (745 to 3038)	1258 (840 to 2166)	1624 (891 to 3234)**	0.40
Low-frequency power (nu)	59.7±16.7	60.5±16.2	58.2±17.7	60.41±16.10	0.99
High-frequency power (nu)	10.9±7.0	11.7±7.6	10.6±6.7	10.55±6.63	0.23
Low to high frequency ratio (log)	6.31 (4.19 to 9.64)	5.83 (1.56 to 3.67)	6.34 (4.07 to 9.83)	6.57 (4.49 to 10.3)	0.19
Orthostatic changes					
Heart rate (beats per minute)	12.4 (11.5 to 13.3)	12.5 (10.9 to 14.1)	12.7 (11.2 to 14.2)	12.0 (10.5 to 13.5)	0.62
Total power (m/s^2^)	0.89 (0.81 to 0.98)	0.98 (0.82 to 1.17)	0.81 (0.68 to 0.97)	0.89 (0.77 to 1.03)	0.43
Low-frequency power (nu)	1.19 (1.14 to 1.24)	1.16 (1.07 to 1.25)	1.18 (1.08 to 1.29)	1.23 (1.13 to 1.33)	0.32
High-frequency power (nu)	0.51 (0.47 to 0.55)	0.56 (0.48 to 0.64)	0.46 (0.40 to 0.53)*	0.50 (0.44 to 0.56)	0.30
Low to high frequency ratio	2.34 (2.16 to 2.56)	2.08 (1.76 to 2.46)	2.53 (2.19 to 2.88)*	2.44 (2.14 to 2.80)	0.13

Values in the supine and standing positions are arithmetic mean (±SD) or geometric mean (IQR). Orthostatic changes in heart rate are reported as the arithmetic mean of the standing minus supine value (95% CI). Orthostatic changes in heart rate variability were computed as the logarithmically transformed standing to supine ratio, for which the geometric mean (95% CI) is given. P values are for linear trend across thirds of the blood lead distribution.

Significance of the difference with the adjacent left column: *P≤0.05; **P≤0.01.

### Peripheral nerve conduction velocity

The peripheral nerve conduction velocity averaged 3.74 (0.53) m/s for the left hand and 3.77 (0.70) m/s for the right hand. Because there was no difference between both sides (p=0.064), in further analyses, we used the mean of both sides, which in all workers averaged 3.74 (0.56). Nerve conduction velocity also did not show any trend across the thirds of the blood lead distribution, averaging 3.78±0.64 m/s, 3.75±0.53 m/s and 3.70±0.49 m/s in the low, medium and high category of blood lead (p value for trend, 0.29).

### Correlations with blood lead

**Figure 2:**
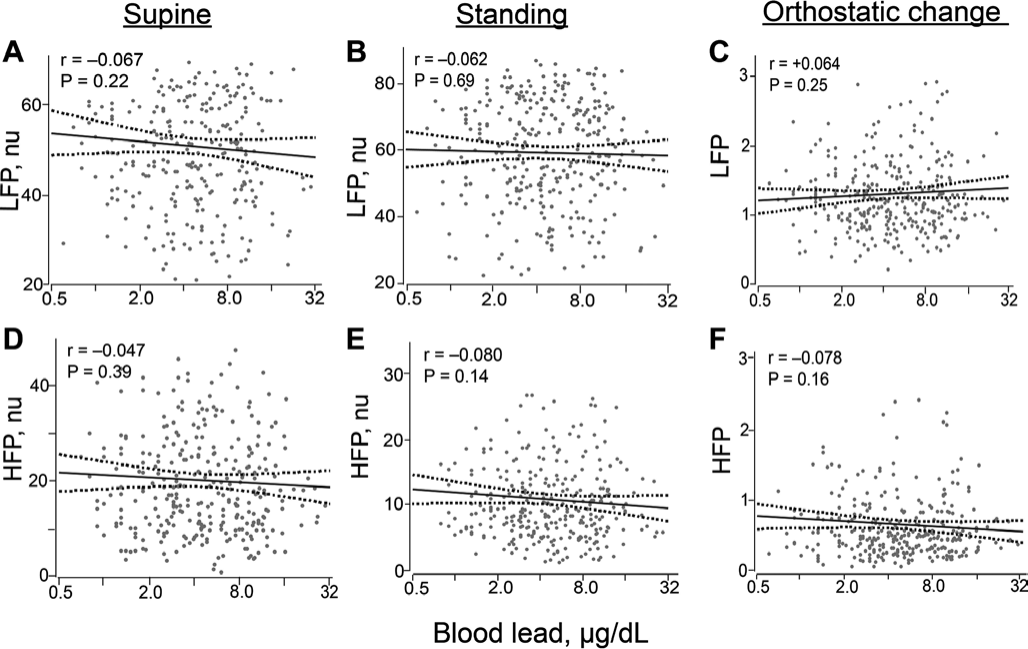
Correlations of blood lead with low-frequency (A–C) and high-frequency (D–F) power in the supine (A, D) and standing (B, E) positions and with the orthostatic changes therein (C, F). For each association the unadjusted regression line with 95% CI is depicted. Adjustment for relevant covariables, identified by stepwise regression, including age, heart rate (or orthostatic change in heart rate), mean arterial pressure and serum insulin did not materially alter these relationships. HFP, high-frequency power; LFP, low-frequency power; nu, normalised unit.

Consistent with the categorical analysis and irrespective of body position, none of the unadjusted correlations of blood lead with heart rate, with the heart rate variability derived by Fourier transform (table 3) or autoregressive modelling (see online supplementary table S2), or orthostatic changes therein reached statistical significance. Figure 2 depicts the correlations for low and high-frequency power. Nerve conduction velocity was also not correlated with blood lead. The difference associated with a 10-fold increase in blood lead was −0.09 m/s (95% CI, −0.26 to 0.08; p=0.29).

**Table 3:** Correlations of blood lead with heart rate variability measured by Fourier transform

Variable	Unadjusted		Adjusted	
	Estimate (95% CI)	P value	Estimate (95% CI)	P value
Supine position				
Total power (%)	23.3 (–9.6 to 68.7)	0.19	3.0 (–20.4 to 33.0)	0.82
Low-frequency power (nu)	–3.00 (–7.83 to 1.83)	0.22	–2.26 (–7.09 to 2.57)	0.36
High-frequency power (nu)	–1.59 (–5.24 to 2.06)	0.39	–2.60 (–6.01 to 0.81)	0.13
Low to high frequency ratio (%)	–1.8 (–20.8 to 21.6)	0.87	6.2(–12.9 to 29.1)	0.56
Standing position				
Total power (%)	11.7 (–17.0 to 50.3)	0.46	–6.0 (–26.2 to 19.7)	0.61
Low-frequency power (nu)	–1.037 (–6.22 to 4.14)	0.69	–1.36 (–6.48 to 3.76)	0.60
High-frequency power (nu)	–1.61 (–3.78 to 0.55)	0.14	–1.66 (–3.79 to 0.47)	0.13
Low to high frequency ratio (%)	14.0 (–7.3 to 40.3)	0.21	–14.0 (–4.9 to 42.2)	0.14
Orthostatic change				
Total power (%)	–8.8 (–31.8 to 17.5)	0.47	–8.8 (–31.8 to 17.5)	0.47
Low-frequency power (%)	4.7 (–6.7 to 20.2)	0.42	–2.3 (–8.8 to 17.4)	0.74
High-frequency power (%)	–8.8 (–27.6 to 14.8)	0.40	–6.7 (–24.2 to 14.8)	0.61
Low to high frequency ratio (%)	14.8 (–8.8 to 47.9)	0.22	7.2 (–14.9 to 34.9)	0.52

Covariables in adjusted models included age, heart rate (or heart rate change for orthostatic changes), mean arterial pressure and serum insulin. Association sizes, given with 95% CI, are the difference in the outcome variable associated with a 10-fold increase in the blood lead concentration. For logarithmically transformed outcomes, differences are given as a percentage or as a percentage change on assuming the standing from the supine position.

Of the covariables considered (see the Methods section), only age, heart rate, mean arterial pressure and serum insulin were identified as covariables of the heart rate variability indices; for nerve conduction velocity, the selected covariables were age, waist to hip ratio, mean arterial pressure and the total to high cholesterol ratio. With adjustment for covariables, none of the correlations of blood lead with heart rate variability, derived by Fourier transform (table 3) or autoregressive modelling (see online supplementary table S2), or with the orthostatic changes therein reached statistical significance. In the multivariable-adjusted analysis, nerve conduction velocity was also not correlated with blood lead. The point estimate associated with a 10-fold increment in blood lead was −0.06 m/s (95% CI, −0.22 to 0.11; p=0.51).

## Discussion

In the newly hired workers enrolled in our current study, the geometric mean blood lead concentration was 4.54 µg/dL. The key findings were that we failed to demonstrate any association of blood lead with the activity of the autonomous nervous system, as captured by heart rate variability, or with peripheral motor nerve conduction velocity at the level of the median nerve.

Among 413 older men (mean age 72.9 years), enrolled in the Veterans Administration Normative Aging Study, patella lead averaged 16.3 µg/g.^7^ With adjustments applied for age, body mass index, fasting glucose, serum lipids, smoking and drinking, antihypertensive drug treatment, room temperature and season, the normalised low and high-frequency power and their ratio were not associated with tibia or patella lead. However, there was a graded, statistically significant reduction in high-frequency power and an increase in low-frequency power and in the low to high frequency ratio in association with higher patella lead as the number of metabolic abnormalities increased from none to 3 or more. The study included 20% of patients with diabetes mellitus, 70% with hypertension and approximately 30% with a history of cardiovascular disease, so this study of older men cannot be extrapolated to the general population. Moreover, the participants were recruited in 1963 and examined from 1991 until 2002,^7^ when according to NHANES data, blood lead levels in the general population in the USA averaged from 2.76 µg/dL (1988–1994)^3^ to 1.64 µg/dL (1999–2002).^4^ Lead is a cumulative toxin and bone lead reflects lifetime rather than recent exposure. The same authors reported 2 years later that the association between heart rate variability and bone lead might be modulated by air pollutants, such as ozone and sulfate, with higher exposure to the air pollutants making study participants more susceptible to cardiac autonomic dysfunction in response to lead exposure.^19^

A cross-sectional study of labourers working in the copper industry in Germany involved 109 clinically healthy exposed male workers (mean age 42.6 years) and 27 non-exposed matched controls, who both underwent psychometric strain testing.^6^ In exposed workers, the blood lead concentration averaged 31.2 µg/dL. Exposed compared with non-exposed participants had a lower heart rate, higher sinus arrhythmia and a dysregulated heart rate variability at rest, during psychometric strain testing and after recovery.^6^ The authors’ interpretation was that long-term lead exposure (19 years) was associated with inhibition of vagal activity,^6^ supporting the authors’ hypothesis that the long vagal nerve, compared with shorter sympathetic fibres, is more vulnerable to the neurotoxic effects of lead. A 4-year follow-up study of 17 exposed workers, during which blood lead increased from 39.4 to 43.3 µg/dL^6^ and a later study with focus on neurocognitive performance^6^ produced confirmatory results. The latter study involved 70 exposed workers whose blood lead at examination averaged 30.4 μg/dL.^6^ However, an earlier study of 98 lead-exposed workers, whose blood lead ranged from 40 to 75 mgμg/dL and who were compared with 85 controls, failed to detect any influence on the autonomic nervous system, as assessed by quantifying sinus arrhythmia.^20^

A meta-analysis of summary statistics, derived from 49 studies for the association between nerve conduction velocity and blood lead, included 1629 controls (women 21.0%) and 2825 exposed individuals (12.9%).^21^ Age and blood lead averaged 39.2 years and 15.7 µg/dL in controls, and 38.4 years and 53.0 µg/dL in exposed individuals.^21^ In exposed individuals compared with controls, conduction velocity was reduced in the median, ulnar and radial nerves in the arm, and in the deep peroneal nerve in the leg. Distal latencies of the median, ulnar and deep peroneal nerves were longer.^20^ No changes in the amplitudes of compound muscle or nerve action potentials were detected. The lowest concentration, at which a relation with blood lead was detected, was 33.0 µg/dL for the nerve conduction velocity of the median sensory nerve.^21^

An association between nerve conduction velocity and blood lead might reflect either a direct neurotoxic effect or an indirect effect mediated by lead-induced dysfunction of other organs, such as the kidney.^21^ In addition, genetic variation (rs1800435) in the δ-aminolevulinic acid dehydratase (*ALAD*) might modify the neurotoxic effects of lead.^21^ Indeed, the second enzyme in the haem biosynthetic pathway, *ALAD* is a homo-octameric protein encoded by a gene localised on human chromosome 9q34.^9^ Expression of the two common alleles, *ALAD*1 and *ALAD*2, results in a polymorphic enzyme system with three distinct isozymes.^9^ Individuals heterozygous or homozygous for the *ALAD*2 allele have significantly higher blood lead levels than do *ALAD*1 homozygotes, when exposed to low or high levels of lead in the environment.^22^ In line with the literature,^22^ among 461 Chinese lead-exposed workers, matched with 175 unexposed controls,^9^ the lead-exposed workers had a higher fraction of the *ALAD*1-2/2-2 genotype than unexposed controls (7.8% vs 2.3%, p=0.01). The lead levels in blood and urine were higher in exposed workers carrying the *ALAD*2 allele compared with *ALAD*1 homozygotes (median blood lead 60.6 vs 49.9 μg/dL; 233 vs 164 μg/g creatinine), while there was no significant difference in the unexposed controls (2.4 vs 3.7 μg/dL and 3.9 vs 6.4 μ g/g creatinine, respectively). High blood and urinary lead were associated with lower sensory and motor conduction velocities in the median, ulnar and peroneal nerves.^9^ This Chinese study suggested that *ALAD* genotypes might modify the changes in peripheral nervous conduction velocity in response to lead exposure.^9^

A detailed description of the potential mechanism of neurotoxicity is beyond the scope of this study. Experimental studies in animals suggested as potential mechanisms: stimulation of the sympathetic preganglionic neurons,^10^ harm to the β-adrenergic system in the brain,^11^ downregulation of cardiac β1-adrenoceptor activity,^14^ upregulated expression of P2X4 receptor in satellite glial cells of the stellate ganglion,^15^ increased sensitivity of chemoreceptor reflex^12^ or decreased sensitivity of the baroreflex.^13^ To what extent these animal experiments can be translated to the human condition remains unclear.

The strong points of our study are that our literature search did not reveal other studies that evaluated the association of autonomous nervous activity or peripheral nerve conduction velocity with blood lead at current exposure levels in humans. Our sample size was of the same order of magnitude as in most previous studies on heart rate variability or peripheral nerve conduction velocity.^12^ Moreover, confounding by drug treatment or previous cardiovascular disease was not an issue in our study. Indeed, only 6.1% of the workers were on antihypertensive drug treatment and only 5.2% had a history of cardiovascular disease (table 1). Nevertheless, our current findings must be interpreted within the context of their limitations. First, findings in workers cannot be extrapolated to the general population, because of the so-called healthy worker effect.^23^ Second, although our study population was ethnically diverse, it included few Asians and no women. Finally, a potential limitation of our study was that we did not measure bone lead as an exposure marker. Approximately 95% of the total body burden of lead is present in the skeleton, and measurement of bone lead levels can provide a more accurate measure of the internal dose.^24^ However, blood lead reflects both recent exogenous exposure and endogenous redistribution of the lead stored in bone.^22^

## Conclusion

In this cross-sectional study, low-level exposure to lead was not associated with changes in autonomous nervous activity, as exemplified by heart rate variability, or with alterations in peripheral nerve conduction velocity. Occupational safety and health regulatory agencies in North America, Europe and Australasia are proposing more stringent workplace limits for lead, but a national expert panel in the USA spent over 2 years discussing whether the current OSHA standards for occupational lead exposure should be tightened without reaching agreement.^25^ SPHERL may provide valuable information on blood lead levels that may not cause adverse health effects associated with occupational lead exposure. The 2-year longitudinal follow-up period built into the study design of SPHERL,^16^ in which blood lead levels are expected to increase from environmental to occupational levels, that is, 20–30 µg/dL, will further inform current and pending workplace limits for lead, many of which are in this blood lead range. This study design will also attempt to resolve the apparent contradiction between general population studies showing associations between adverse health effects with blood lead levels below 10 µg/dL^26^ and studies conducted in occupational cohorts, in whom adverse effects of lead exposure occur at much higher blood lead levels.^23^
