# Supplementary material for: Heart rate variability and peripheral nerve conduction velocity in relation to blood lead in newly hired lead workers
Source: Occup Environ Med. 2019 Mar 30;76(6):382–8. doi: 10.1136/oemed-2018-105379 (PMC6585574; doi:10.1136/oemed-2018-105379)
Supplement: Supplementary file 1 [file oemed-2018-105379supp001.doc]

**OCCUPATIONAL AND ENVIRONMENTAL MEDICINE**

**Supporting information**

HEART RATE VARIABILITY AND PERIPHERAL NERVE CONDUCTION VELOCITY IN RELATION TO BLOOD LEAD IN WORKERS PRIOR TO CHRONIC OCCUPATIONAL EXPOSURE

Cai-Guo Yu, Fang-Fei Wei, Wen-Yi Yang, Zhen-Yu Zhang, Blerim Mujaj, Lutgarde Thijs, Ying-Mei Feng, Jan A. Staessen

**Table of contents**

Quality assurance and quality control of the biochemical methods p2

References P3

**Table S1** Heart rate variability by autoregressive modelling p4

**Table S2** Correlations of blood lead with heart rate variability as measured by
autoregressive modelling p5

**Figure S1** Bland–Altman plot for nerve conduction velocity p6

**Quality assurance and quality control of the biochemical methods**

The accuracy of the lead tests was verified by use of proficiency samples purchased from the College of American Pathologists (CAP) and the Pennsylvania Department of Blood Lead Programs.^1^ Proficiency testing was performed in six separate trial runs, including in total 30 test samples annually. All survey materials were handled in the same manner as the study samples and processed with the normal workflow utilizing the same repeat/dilution protocols and calibration and quality control frequency.^1^ Compliance with Clinical Laboratory Improvement Amendments (CLIA), CAP and New York State accreditation and regulatory requirements was verified routinely with test level review of the laboratory services by external auditors. Calibrators with certified accuracy (National Institute of Standards and Technology [www.nist.gov]) were included in each batch of study samples and spanned the range of the analytical measurement range. Accuracy was evaluated on Westgard Rules^2^ and defined within the total allowable error established with review of the CAP, Centers for Disease Control and Prevention, CLIA 88,^3^ and OSHA guidelines. Accuracy, defined as the deviation from known lead standards ran along with the study samples, was within 10%.^1^ The bias determined according to the Bland and Altman approach^4^ in 30 spilt blood samples with blood lead concentrations (average in duplicate samples) ranging from 0.70 to 27.9 μg/dL, was 0.08 μg/dL (95% confidence interval [CI], ‑0.01 to 0.18, P=0.078). The repeatability coefficient, defined as twice the SD of the signed differences between duplicate measurements,^4^ was 0.52. Expressed as a percentage of the mean blood lead concentration or as a percentage of near maximal variation in blood lead (four times the SD of the logarithmically transformed distribution), the repeatability coefficient was 6.7% and 1.9%, respectively. Lower values indicate better repeatability. Over three evaluations, the laboratory obtained a proficiency score of 100% for blood lead, 100% for routine biochemistry, and 98% for serum insulin.

**References**

1 Centers for Disease Control and Prevention. Screening young children for lead poisoning: Guidance for state and local public health officials; Appendix C1: The Lead Laboratory. Accessed 18 December, 2018.

2 Westgard JO, Barry PL, Hunt MR*, et al.* A multi-rule Shewhart chart for quality control in clinical chenistry. *Clin Chem* 1981;27:493-501.

3 Centers for Disease Control and Prevention. CLIA '88 Focus on Clinic and Office Laborarories. Accessed 18 December, 2018.

4 Bland JM, Altman DG. Statistical methods for assessing agreement between two methods of clinical measurement. *Lancet* 1986;1:307-10.

**Table S1**

**Heart rate variability by autoregressive modelling**

| **Characteristic** | **All workers** |  | **Stratified by thirds of the blood lead distribution** | | | |
| --- | --- | --- | --- | --- | --- | --- |
|  |  |  | **<3.1 μg/dL** | **3.1–7.0 μg/dL** | **>7.0 μg/dL** | **P value** |
| Number in category | 328 |  | 106 | 112 | 110 |  |
| Supine position |  |  |  |  |  |  |
| Heart rate (beats/minute) | 65.4±10.8 |  | 66.9±11.3 | 65.2±10.2 | 64.3±10.6 | 0.080 |
| Total power (ms2) | 923 (481 to 1702) |  | 927(496 to 1916) | 873(450 to 1548) | 972(500 to 1816) | 0.70 |
| Low frequency power (nu) | 50.6±14.4 |  | 52.50±14.91 | 49.63±13.81 | 49.65±14.50 | 0.15 |
| High frequency power (nu) | 21.7±11.7 |  | 21.00±11.51 | 23.23±12.84 | 20.78±10.56 | 0.88 |
| Low-to-high frequency ratio | 2.61 (1.66 to 4.26) |  | 2.79(1.76 to 4.64) | 2.44(1.35 to 3.95) | 2.62(1.66 to 3.94) | 0.49 |
| Standing position |  |  |  |  |  |  |
| Heart rate (beats/minute) | 77.8±12.7 |  | 79.4±13.5 | 78.0±12.5 | 76.3±12.0 | 0.069 |
| Total power (ms2) | 869 (479 to 1612) |  | 879(452 to 1609) | 778(475 to 1460) | 961(564 to 1868) | 0.48 |
| Low frequency power (nu) | 61.6±14.6 |  | 62.85±14.23 | 58.82±15.80 * | 63.31±13.44 * | 0.80 |
| High frequency power (nu) | 11.3±7.2 |  | 11.89±7.85 | 10.27±6.05 | 11.87±7.61 | ﹥0.99 |
| Low-to-high frequency ratio (log) | 6.23 (4.11 to 9.75) |  | 6.04(4.08 to 9.48) | 6.49(4.15 to 10.17) | 6.16(4.13 to 9.52) | 0.83 |
| Orthostatic changes |  |  |  |  |  |  |
| Heart rate (beats per minute) | 12.4 (11.5 to 13.3) |  | 12.5 (10.9 to 14.1) | 12.7 (11.2 to 14.2) | 12.0 (10.5 to 13.5) | 0.98 |
| Total power ms^2^ | 0.94 (0.86 to 1.03) |  | 0.95(0.81 to 1.11) | 0.89(0.76 to 1.04) | 0.99(0.87 to 1.12) | 0.69 |
| Low-frequency power (nu) | 1.24 (1.19 to 1.29) |  | 1.22(1.14 to 1.31) | 1.18(1.10 to 1.28) | 1.31(1.22 to 1.40) | 0.19 |
| High-frequency power (nu) | 0.51 (0.48 to 0.55) |  | 0.56(0.48 to 0.64) | 0.44(0.39 to 0.50) ** | 0.55(0.49 to 0.62) * | 0.88 |
| Low-to-high frequency ratio | 2.39 (2.21 to 2.60) |  | 2.16(1.85 to 2.54) | 2.66(2.34 to 3.01) * | 2.36(2.06 to 2.69) | 0.42 |

Values in the supine and standing position are arithmetic mean (± SD) or geometric mean (interquartile range). Orthostatic changes in heart rate are reported as the arithmetic mean of the standing minus supine value (95% confidence interval). Orthostatic changes in heart rate variability were computed as the logarithmically transformed standing-to-supine ratio, for which the geometric mean (95% confidence interval) is given. P‑values are for linear trend across thirds of the blood lead distribution. Significance of the difference with the adjacent left column: * P≤0.05; ** P≤0.01.

**Table S2**

**Correlations of blood lead with heart rate variability measured by autoregressive modelling**

| **Variable** |  | **Unadjusted** | |  | **Adjusted** | |  |
| --- | --- | --- | --- | --- | --- | --- | --- |
|  |  | **Estimate (95% CI)** | **P** |  | **Estimate (95% CI)** | **P value** |  |
| Supine position |  |  |  |  |  |  |  |
| Total power (%) |  | 6.4 (–20.2 to 41.9) | 0.67 |  | –11.7 (–29.7 to 10.7) | 0.28 |  |
| Low-frequency power (nu) |  | –2.75 (–7.22 to 1.72) | 0.23 |  | –1.99 (–6.41 to 2.43) | 0.38 |  |
| High-frequency power (nu) |  | –1.29 (–4.92 to 2.35) | 0.49 |  | –2.43 (–5.83 to 0.96) | 0.16 |  |
| Low-to-high frequency ratio (%) |  | –2.5 (–20.6 to 20.2) | 0.82 |  | 5.7 (–12.9 to 28.8) | 0.58 |  |
| Standing position |  |  |  |  |  |  |  |
| Total power (%) |  | 10.2 (–18.2 to 47.9) | 0.52 |  | –9.4 (–27.9 to 13.8) | 0.61 |  |
| Low-frequency power (nu) |  | –0.22 (–4.32 to 4.77) | 0.92 |  | –0.09 (–4.50 to 4.32) | 0.60 |  |
| High-frequency power (nu) |  | –0.16 (–2.40 to 2.09) | 0.89 |  | –0.38 (–2.57 to 1.81) | 0.13 |  |
| Low-to-high frequency ratio (%) |  | 3.6 (–16.4 to 25.9) | 0.81 |  | 5.9 (–12.9 to 28.8) | 0.14 |  |
| Orthostatic change |  |  |  |  |  |  |  |
| Total power (%) |  | 2.3 (–18.7 to 31.8) | 0.78 |  | 1.2 (–20.0 to 27.9) | 0.92 |  |
| Low-frequency power (%) |  | 7.2 (–4.5 to 20.2) | 0.29 |  | 4.7 (–6.7 to 17.5) | 0.45 |  |
| High-frequency power (%) |  | 2.3 (–16.8 to 25.9) | 0.83 |  | 4.7 (–14.9 to 25.9) | 0.72 |  |
| Low-to-high frequency ratio (%) |  | 4.7 (–16.8 to 31.8) | 0.67 |  | 1.6 (–18.7 to 25.9) | 0.89 |  |

Adjusted models included as covariables age, heart rate (or heart rate change for orthostatic changes), mean arterial pressure and serum insulin. Association sizes, given with 95% confidence interval, express the difference in the outcome variable associated with a 10‑fold increase in the blood lead concentration. For logarithmically transformed outcomes, differences are given as a percentage or as a percentage change on assuming the standing from the supine position.


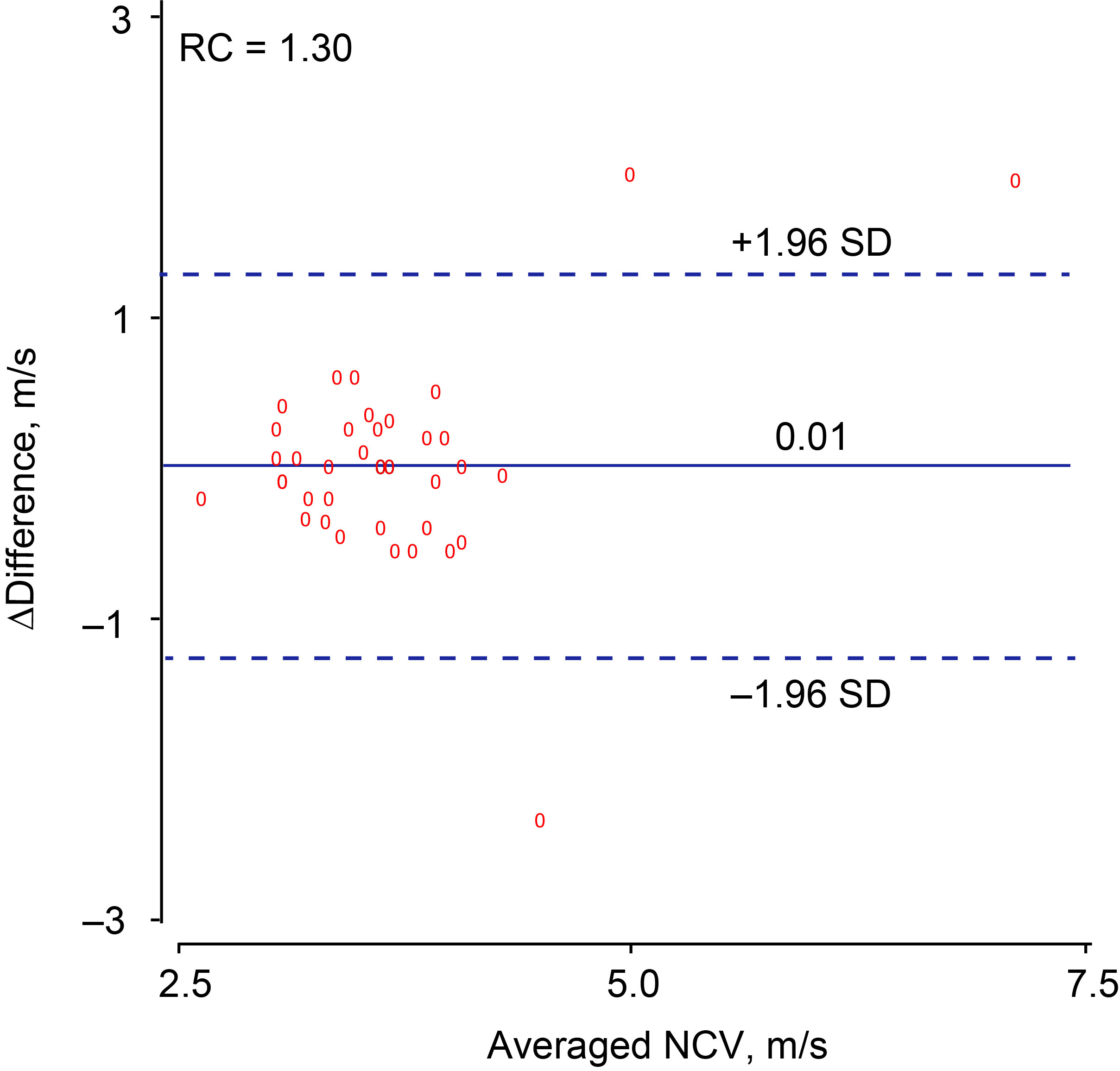


**Figure S1**

Bland–Altman plot for nerve conduction velocity (NCV) as measured at the right and left side in 40 randomly selected workers. The difference (Δ) between both measurements (right minus left side) was plotted against the average of both measurements. The bias was 0.01 m/s (p=0.90). The reproducibility coefficient (RC) is twice the SD of the signed differences between duplicate measurements (Bland JM, Altman DG. *Lancet* 1986;i [issue 8476]:307-10).
